# *Euodia daniellii* Hemsl. Extract and Its Active Component Hesperidin Accelerate Cutaneous Wound Healing via Activation of Wnt/β-Catenin Signaling Pathway

**DOI:** 10.3390/molecules27207134

**Published:** 2022-10-21

**Authors:** Minguen Yoon, Seol Hwa Seo, Seonghwi Choi, Gyoonhee Han, Kang-Yell Choi

**Affiliations:** 1 Department of Biotechnology, College of Life Science and Biotechnology, Yonsei University, Seoul 03722, Korea; 2 Department of Interdisciplinary Program of Integreated OMICS for Biomedical Sciences, Yonsei University, Seoul 03722, Korea; 3 CK Regeon Inc., Seoul 03722, Korea

**Keywords:** *Euodia daniellii* Hemsl. extract, hesperidin, human keratinocyte, human dermal fibroblast, cutaneous wound healing, Wnt/β-catenin signaling pathway

## Abstract

The activation of the Wnt/β-catenin signaling pathway plays a key role in the wound-healing process through tissue regeneration. The extract of *Euodia daniellii* Hemsl. (*E. daniellii*), a member of the Rutaceae family, activates the Wnt/β-catenin signaling pathway. However, the function of *E. daniellii* in wound healing has not yet been elucidated. We performed a migration assay to determine the wound-healing effect of *E. daniellii* extract in vitro using human keratinocytes and dermal fibroblast. In addition, a mouse acute wound model was used to investigate the cutaneous wound-healing effect of *E. daniellii* extract in vivo and confirm the potential mechanism. *E. daniellii* extract enhanced the migration of human keratinocytes and dermal fibroblasts via the activation of the Wnt/β-catenin pathway. Moreover, the *E. daniellii* extract increased the levels of keratin 14, PCNA, collagen I, and α-SMA, with nuclei accumulation of β-catenin in vitro. *E. daniellii* extract also efficiently accelerated re-epithelialization and stimulated wound healing in vivo. Furthermore, we confirmed that hesperidin, one of the components of *E. daniellii*, efficiently accelerated the migration of human keratinocytes and dermal fibroblasts, as well as wound healing in vivo via the activation of the Wnt/β-catenin pathway. Overall, *E. daniellii* extract and its active component, hesperidin, have potential to be used as therapeutic agents for wound healing.

## 1. Introduction

Wound healing is the process of restoring the structure and function of damaged tissues through the homeostasis, inflammatory, proliferative, and remodeling phases [1]. These processes are orchestrated by the interactions between multiple cells, growth factors, cytokines, etc. [2]. The failure of this delicate process results in chronic wound or abnormal scar formation [3]. With a growing global market and medical needs, numerous wound care products have been developed [4]. However, traditional therapies, such as dressing or bandages protecting wounds and antibiotics to prevent infection, have limitations [5]. Recently developed therapies using growth factors are also limited in practical use because of their high cost, low delivery rates, and poor efficacies [6,7]. Considering the limitations of current wound-healing agents, alternative agents are needed to overcome the hurdle of current therapies.

Over the past several decades, significant improvements have been made in understanding the relationships between signaling pathways and the wound-healing process [8]. Among them, the Wnt/β-catenin signaling pathway plays a major role in the wound-healing process [9,10]. The Wnt/β-catenin signaling pathway determines the fate and proliferation of progenitor cells during embryonic development and regulates tissue homeostasis during the postnatal period [11,12]. Moreover, the Wnt/β-catenin signaling pathway plays an important role in the regulation of adult stem cells and tissue regeneration during wound healing [8]. Therefore, the Wnt/β-catenin signaling pathway may be an ideal target for the development of wound-healing agents [13,14].

Plant extracts, including herbal medicinal agents, are relatively safe natural agents that have often been used as therapeutics. Herbal medicines are reported to provide a moist environment, lower infection, and prevent scar formation in wound management [15]. Additionally, plant extracts cause less irritation, sensitization, and toxicity to the wound area [16]. *E. daniellii*, a member of the Rutaceae family, has been used as a herbal medicine for the treatment of dermatitis, headache, and gastric inflammation [17]. It is also known that *E. daniellii* extract stimulates osteoblast differentiation via the activation of the Wnt/β-catenin signaling pathway [18]. However, the role of *E. daniellii* in wound healing has not been reported.

Here, we investigated the wound-healing ability of *E. daniellii* extract. Because *E. daniellii* extract is known to activate Wnt/β-catenin signaling, we first tested its role in the migration of human keratinocytes and dermal fibroblasts to investigate its role in wound healing. We also detected wound-healing marker changes in human keratinocytes and dermal fibroblasts to confirm the potential mechanism. Furthermore, we tested the in vivo wound-healing effect of *E. daniellii* extract, including neo-epidermis formation, using a a mouse acute wound model [9]. Finally, we tested the wound-healing effects of components of *E. daniellii* extract by using identical in vitro and in vivo systems.

## 2. Materials and Methods

### 2.1. Preparation of E. daniellii Extract

*E. daniellii* was obtained from the Korea Plant Extract Bank of the Korea Research Institute of Bioscience and Biotechnology (Daejeon, Korea). The plant was collected from Boryeong-si, Chungcheongnam-do, KOREA in 2007. A voucher specimen (KRIB 0014366) was stored in the herbarium of the Korea Research Institute of Bioscience and Biotechnology. The *E. daniellii* plant (170 g), dried in the shade and powdered, was added to 1 L of methyl alcohol 99.9% (HPLC grade) and was extracted through 30 cycles (40 KHz, 1500 W, 15 min ultrasonication-120 min standing per cycle) at room temperature using an ultrasonic extractor (SDN-900H, SD-ULTRASONIC CO., LTD, Seoul, Korea). After filtration (Qualitative Filter No.100, HYUNDAI MICRO Co., LTD, Ansung, Korea) and drying under reduced pressure, *E. daniellii* extract (15.91 g) was obtained.

### 2.2. Components of E. daniellii Extract

The components of *E. daniellii*, hesperidin, vitexin, limonin, myo-inositol, and uracil were purchased from Sigma-Aldrich (St. Louis, MO, USA) and were dissolved in dimethyl sulfoxide (DMSO; Sigma Aldrich) before use.

### 2.3. Cell Culture and In Vitro Wound-Healing Assay

HaCaT keratinocytes and human dermal fibroblasts were cultured in Dulbecco’s modified Eagle’s medium (DMEM; Gibco, Grand Island, NY, USA) containing 10% (*v*/*v*) heat-inactivated fetal bovine serum (FBS; Gibco), 100 mg/mL of penicillin (Gibco), and 100 mg/mL of streptomycin (Gibco), at 37 °C in a humidified incubator with 5% (*v*/*v*) CO_2_. For the in vitro wound-healing assay, HaCaT keratinocytes or human dermal fibroblasts were seeded in 12-well plates in DMEM containing 10% FBS in triplicate at a density of 4 × 10^5^ cells/well. After a 24 h attachment period, the monolayers were scratched with a sterile pipette tip and were incubated with a medium containing 5% FBS with or without *E. daniellii* extract (1 or 5 μg/mL), individual *E. daniellii* components (5 μM), or VPA (100 μM). After 24 h, the cells were harvested, washed once with cold phosphate-buffered saline (PBS, pH 7.4), fixed in 4% paraformaldehyde (PFA) for 15 min at room temperature, and stained with 2% (*w*/*v*) crystal violet. The wound-closure area was measured using the NIS-Elements imaging software (Nikon, Tokyo, Japan) (*n* = 3).

### 2.4. Transwell Migration Assay

Transwell migration assays were performed in matrix-coated transwell plates (8 μm pore size; Corning Life Sciences, Lowell, MA, USA), as described in our previous study [19]. Filters were coated with bovine serum albumin (BSA) (100 μg/mL), vitrogen, and fibronectin (10 μg/mL) in PBS for 1 h at 37 °C. HaCaT keratinocytes and human dermal fibroblasts were seeded onto the filters at a density of 5 × 10^4^ cells/well, and different doses of *E. daniellii* extract (1 or 5 μg/mL), individual components (5 μM), or VPA (100 μM) were added to the upper and lower compartments prior to seeding the cells. After 24 h of incubation, the cells in the inner chamber were removed, and cells on the outer surface were fixed with 4% paraformaldehyde (PFA) and were stained with 2% (*w*/*v*) crystal violet. Migrating cells were visualized using a bright-field optical microscope (Nikon TE-200U), and the migrated areas were measured using NIS-Elements imaging software (Nikon, Tokyo, Japan) (*n* = 3).

### 2.5. Luciferase Assay

HEK293-TOP cells were seeded into 96-well plates at a density of 2.5 × 10^4^ cells/well and were incubated in a medium containing 10% FBS for 24 h. The cells were incubated for 24 h with or without 1, 5, 10, 25, 50 μg/mL of *E. daniellii* extract or 1, 5, 10, 25, 50 μM of each of its components (Sigma-Aldrich). The total cell lysates were extracted with 25 μL of 1× reporter lysis buffer (Promega, Madison, WI, USA) per well, and luciferase activity was measured using a microplate luminometer (BMG Labtech, Offenburg, Germany).

### 2.6. Western Blot Analysis

Cells and tissues were ground and lysed in a RIPA buffer (150 mM NaCl, 10 mM Tris, pH 7.2, 0.1% SDS, 1.0% Triton X-100, 1% sodium deoxycholate, and 5 mM EDTA). The samples were separated on 10–12% SDS polyacrylamide gels and were transferred onto PROTRAN nitrocellulose membranes (Schleicher and Schuell Co., New York, NY, USA). After blocking with PBS containing 5% non-fat dry skim milk and 0.07% (vol/vol) Tween 20, the membranes were incubated with antibodies against β-catenin (1:1000; Santa Cruz Biotechnology, Inc., Dallas, TX, USA), α-SMA (1:1000; Abcam, Cambridge, UK), keratin 14 (1:1000; Covance, Durham, NC, USA), collagen I (1:1000; Abcam), PCNA (1:500; Santa Cruz Biotechnology), α-tubulin (1:5000; Oncogene Research Products, San Diego, CA, USA), or Erk (1:5000; Cell Signaling Technology, Danvers, MA, USA) at 4 °C overnight. The samples were then incubated with a horseradish peroxidase-conjugated antirabbit (1:5000; Bio-Rad Laboratories, Hercules, CA, USA) or antimouse IgG secondary antibody (1:5000; Cell Signaling Technology). The protein bands were visualized using an enhanced chemiluminescence kit (Amersham Bioscience, Piscataway, NJ, USA) and a luminescent image analyzer, LAS-4000 (Fujifilm, Tokyo, Japan).

### 2.7. β-Catenin Knockdown by Small Interfering RNA Transfection

The human β-catenin small interfering RNA (siRNA) target sequences were 5′-GAAACGGCTTTCAGTTGAG-3′ and 5′-AAACTACTGTGGACCACAAGC-3′. β-catenin siRNA was transfected into HaCaT keratinocytes or human dermal fibroblasts using the Lipofectamine Plus reagent (Invitrogen, Carlsbad, CA, USA) at a final concentration of 100 nM. The cells were scratched and incubated with or without *E. daniellii* extract (1 or 5 μg/mL), individual components (1 or 5 μM), or VPA (100 μM). β-catenin knockdown was confirmed by immunoblotting analysis, as described earlier.

### 2.8. Immunocytochemistry

HaCaT keratinocytes and human dermal fibroblasts were plated in 12-well culture plates. The cells were washed once in PBS, fixed with 4% PFA in PBS for 15 min at room temperature, and permeabilized in 0.1% Triton X-100 for 30 min at room temperature. After blocking with 5% BSA for 30 min at room temperature, the cells were incubated with primary antibodies against β-catenin (1:100; BD Transduction Laboratories, Lexington, KY, USA), phalloidin (1:200; Molecular Probes, Eugene, OR, USA), or collagen I (1:100; Abcam) overnight at 4 °C. The cells were washed with PBS and incubated with Alexa Fluor 488-conjugated or Alexa Fluor 555-conjugated IgG secondary antibody (1:400; Molecular Probes, Eugene, OR, USA) for 1 h at room temperature, counterstained with 4′,6-diamidino-2-phenylindole (DAPI; 1:5000; Boehringer Mannheim, Mannheim, Germany), and examined under a confocal microscope (LSM510 META; Carl Zeiss, Gottingen, Germany).

### 2.9. Animals and In Vivo Wound-Healing Assay

Seven-week-old male C3H mice were purchased from Orient Bio Co. (Seongnam-si, Korea) and were allowed to adapt to their new environment for one week. The procedures were reviewed and approved by the Institutional Animal Care and Use Committee (IACUC) of the Yonsei Laboratory Animal Research Center (IACUC-A-201609-407-01, IACUC-A-201705-197-01). The animals were maintained under a 12 h light/12 h darkness cycle at 22–25 °C under conventional conditions and were fed with a standard rodent chow diet and water. To determine the therapeutic potential of the *E. daniellii* extract and hesperidin on wound healing, 8-week-old C3H mice were anesthetized, the dorsal hair was removed using hair clippers, the skin was cleaned with 70% ethanol, and full-thickness 1.0 cm^2^ dorsal wounds were made on the backs of the mice. *E. daniellii* extract (1 or 5 mg/mL), hesperidin (1 or 5 mM), valproic acid (VPA: 500 mM, for positive control), or epidermal growth factor (EGF: 100 μM, for positive control) was topically applied daily until wound closure (*n* = 6). The wound sizes were measured every other day, under the assumption that the wound depths in each animal were almost constant. Wounded skin tissue samples were harvested and evaluated using immunohistochemistry (IHC) analysis.

### 2.10. Immunohistochemical Analysis

The tissues were fixed with 4% PFA and paraffin-embedded tissues were sectioned into 4 μm thickness. The slides were deparaffinized in xylene and were rehydrated using a graded dose of alcohol. For antigen retrieval, the slides were autoclaved in 110 mM sodium citrate buffer. Sections were pre-incubated in PBS and then blocked by using PBS containing 5% BSA and 1% goat serum for 30 min at room temperature. Tissue sections were incubated overnight at 4 °C with primary antibodies against β-catenin (1:100; BD Transduction Laboratories), proliferating cell nuclear antigen (1:500; PCNA, Santa Cruz Biotechnology), keratin 14 (1:500; Covance), or collagen I (1:100; Abcam). The sections were rinsed with PBS, incubated with an IgG secondary antibody conjugated to Alexa Fluor 488 or Alexa Fluor 555 (1:400; Molecular Probes) for 1 h at room temperature, and counterstained with DAPI (1:5000; Boehringer Mannheim). Fluorescent signals were visualized using a LSM510 META confocal microscope (Carl Zeiss). For hematoxylin and eosin (H&E) staining, the sections were stained with hematoxylin for 5 min and eosin for 1 min. The slides were then dehydrated using a graded dose of alcohol series, cleared in xylene, and mounted in Permount (Fisher Scientific, Waltham, MA, USA). H&E-stained tissue sections were visualized using a bright-field optical microscope (Nikon TE-200U).

### 2.11. CellTiter-Glo Luminescent Cell Viability Assay

HaCaT keratinocytes and human dermal fibroblasts were plated at a density of 1 × 10^5^ cells/well in a 24-well plate. The cells were then treated with DMSO, a gradient dose of *E. daniellii* extract, or hesperidin for 24 h. Cell viability was assessed using the CellTiter-Glo mixture, as recommended by the supplier. Adenosine triphosphate (ATP) was quantified spectrophotometrically at 560 nm using a microplate luminometer (BMG Labtech, Ortenberg, Germany).

### 2.12. High-performance liquid chromatography Analysis

The composition of vitexin, uracil and heperidin in E. daniellii was calculated based on UV absorbance (254 nm) peak area obtained through high-performance liquid chromatography (HPLC). HPLC analysis was performed on Shimadzu HPLC instrument (Shimadzu, Kyoto, Japan) using Agilent ZORBAX Eclipse Plus C18 column (95Å, 4.6 × 150 mm, 5 μm), mobile phase; acetonitrile and water, both containing 0.05% formic acid.

### 2.13. Statistical Analysis

Statistical analyses were performed using unpaired two-tailed Student’s *t*-test. Asterisks indicate statistically significant differences, with one asterisk indicating *p* < 0.05 and two asterisks indicating *p* < 0.005.

## 3. Results and Discussion

### 3.1. E. daniellii Extract Enhances the Migration of Human Keratinocytes and Dermal Fibroblasts via Activation of the Wnt/β-Catenin Signaling Pathway

To characterize the role of *E. daniellii* extract on wound healing in vitro, we first confirmed the Wnt/β-catenin signaling activation characteristics of the *E. daniellii* extract using the TOPflash reporter assay, and *E. daniellii* extract activated Wnt/β-catenin signaling as revealed [18] (Figure S1A). We used both HaCaT keratinocytes and human dermal fibroblasts, as they are essential for the maintenance of skin homeostasis and cutaneous wound healing [20]. The *E. daniellii* extract did not show any significant cellular toxicity up to a concentration of 50 μg/mL, as confirmed by tests using human keratinocytes, dermal fibroblasts, and fragile primary neural stem cells (Figure S1B,C).

**Figure 1:**
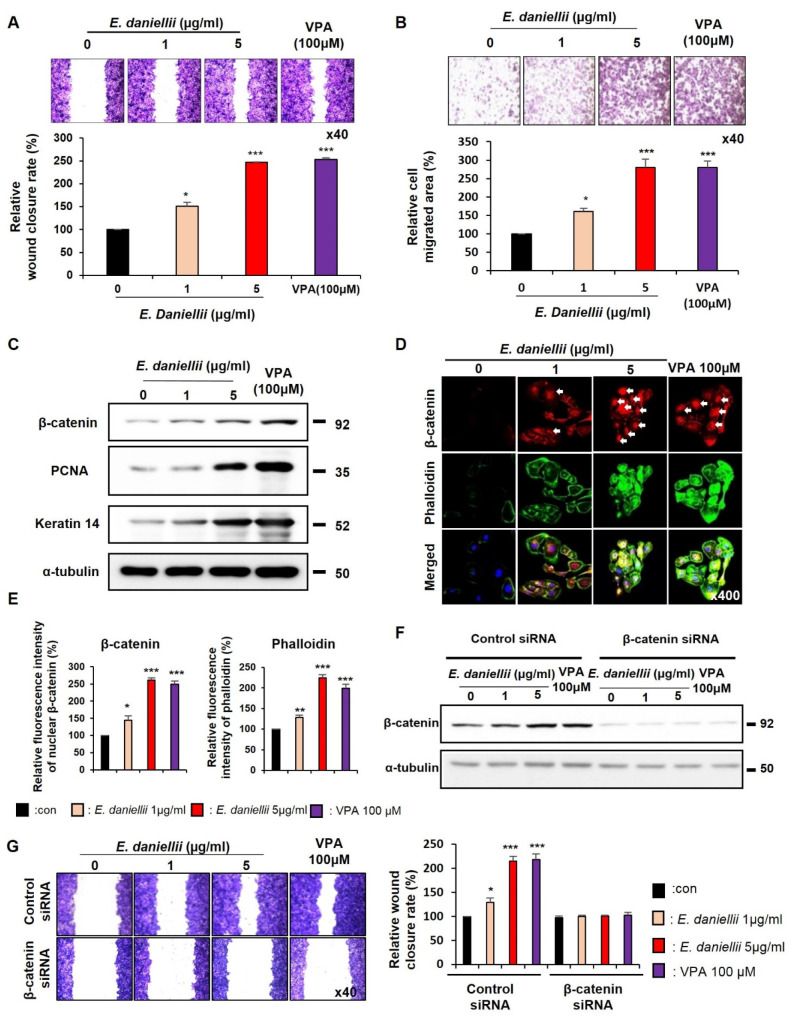
Effects of *E. daniellii* extract on migration of human keratinocytes. (**A**–**D**) HaCaT keratinocytes were treated with vehicle (0.1% [*v*/*v*] DMSO), *E. daniellii* extract (1, 5 μg/mL), or VPA (100 μM) for 24 h. (**A**,**B**) The in vitro wound-healing and transwell assays were performed as described in the Materials and Methods section. (**A**) Representative images of in vitro wound-healing assay (upper) and quantitative measurement of relative wound-closure rate (lower). (**B**) Representative images of transwell assay and quantitative measurement of the area of migrated cells. (**C**) Immunoblotting analysis data. (**D**) ICC staining for β-catenin and total phalloidin. Nuclei were counterstained with DAPI. Arrows indicate nucleus-localized β-catenin. (**E**) Quantitative measurements of intensities of β-catenin and phalloidin for the data from the experiments in Figure 1D (*n* = 12 taken in three different images). (**F**,**G**) HaCaT keratinocytes were transfected with β-catenin siRNA or negative control for 12 h. After transfection, HaCaT keratinocytes were treated with vehicle (0.1% DMSO), *E. daniellii* extract (1, 5 μg/mL), or VPA (100 μM) for 24 h. (**F**) Immunoblotting analysis data for transfected keratinocytes. (**G**) Representative images of in vitro wound-healing assay of transfected keratinocytes and quantitative measurement of wound-closure rate. Original magnification: (**A**,**B**), ×40; (**D**), ×400. Values are expressed as means ± SEM. * *p* < 0.05; ** *p* < 0.005; *** *p* < 0.0005, significantly different from vehicle, control, or as indicated.

**Figure 2:**
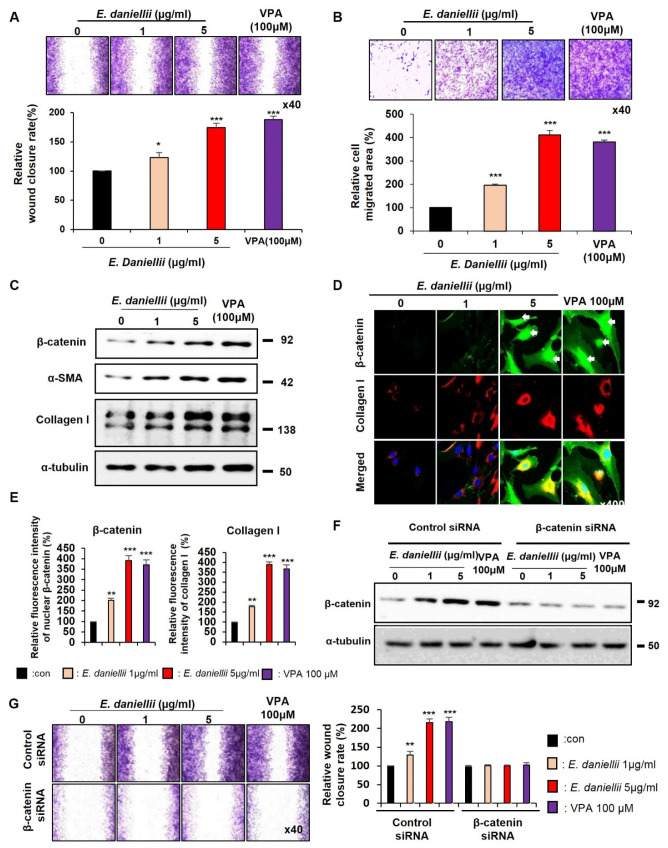
Effects of *E. daniellii* extract on migration of human dermal fibroblasts. (A–D) Human dermal fibroblasts were treated with vehicle (0.1% DMSO), *E. daniellii* extract (1, 5 μg/mL), or VPA (100 μM) for 24 h. (**A**,**B**) The in vitro wound-healing and transwell assays were performed as described in the Materials and Methods section. (**A**) Representative images of in vitro wound-healing assay and quantitative measurement of relative wound-closure rate. (**B**) Representative images of transwell assay and quantitative measurement of relative cell-migrated area. (**C**) Immunoblotting analysis data. (**D**) ICC staining for β-catenin and collagen I. Nuclei were counterstained with DAPI. Arrows indicate nucleus-localized β-catenin. (**E**) Quantitative measurements of intensities of β-catenin and Collagen I for the data from the experiments in Figure S2D (*n* = 10 taken in three different images). (**F**,**G**) Fibroblasts were transfected with β-catenin siRNA or negative control for 12 h. After transfection, fibroblasts were treated with vehicle (0.1% DMSO), *E. daniellii* extract (1, 5 μg/mL), or VPA (100 μM) for 24 h. (**F**) Immunoblotting analysis data for transfected fibroblasts. (**G**) Representative images of in vitro wound-healing assay of transfected fibroblasts and quantitative measurement of wound-closure rate. Original magnification: (**A**,**B**), ×40; (**D**), ×400. Values are expressed as means ± SEM. * *p* < 0.05; ** *p* < 0.005; *** *p* < 0.0005, significantly different from vehicle, control, or as indicated.

The migration of keratinocytes and fibroblasts was dose-dependently increased by the *E. daniellii* extract treatment, as shown by both the in vitro wound-healing and transwell assays (Figure 1A,B and Figure 2A,B). The relative wound-closure rate and cell migration area revealed by the treatment with 5 μg/mL *E. daniellii* extract were equivalent to those observed after treatment with 100 μM VPA, a known Wnt/β-catenin signaling activator [13] (Figure 1A,B and Figure 2A,B). We have also confirmed that 5 μg/mL and/or above of *E. daniellii* extract had similar wound healing effect in HaCaT cells compared to positive control VPA 100μM (Figure S1D).

To determine whether the *E. daniellii* extract accelerates wound healing by activating Wnt/β-catenin signaling in vitro, we first examined the dose-dependent effects of *E. daniellii* extract on the levels of β-catenin in human keratinocytes and fibroblasts. Treatment with the *E. daniellii* extract dose-dependently increased β-catenin levels with an increase in keratin 14 in keratinocytes, α-smooth muscle actin (α-SMA), collagen I, and the proliferation marker proliferating cell nuclear antigen (PCNA) in fibroblasts (Figure 1C and Figure 2C). The upregulation of Wnt/β-catenin signaling and wound-healing markers was further confirmed by immunocytochemistry (ICC) analyses of human keratinocytes and fibroblasts treated with *E. daniellii* extract (Figure 1D and Figure 2D). Upon treatment with *E. daniellii* extract, nuclear-localized active β-catenin was critically and dose-dependently increased with the increment of phalloidin, stress fibers, and cortical networking markers, as well as collagen I in human keratinocytes and fibroblasts (Figure 1E and Figure 2E). However, the wound-healing capability of *the E. daniellii* extract was completely abolished by β-catenin knockdown (Figure 1F,G and Figure 2F,G).

These results show that *E. daniellii* extract increases the migration and in vitro wound healing of keratinocytes and fibroblasts via the activation of the Wnt/β-catenin pathway.

### 3.2. E. daniellii Extract Promotes Cutaneous Wound Healing In Vivo

**Figure 3:**
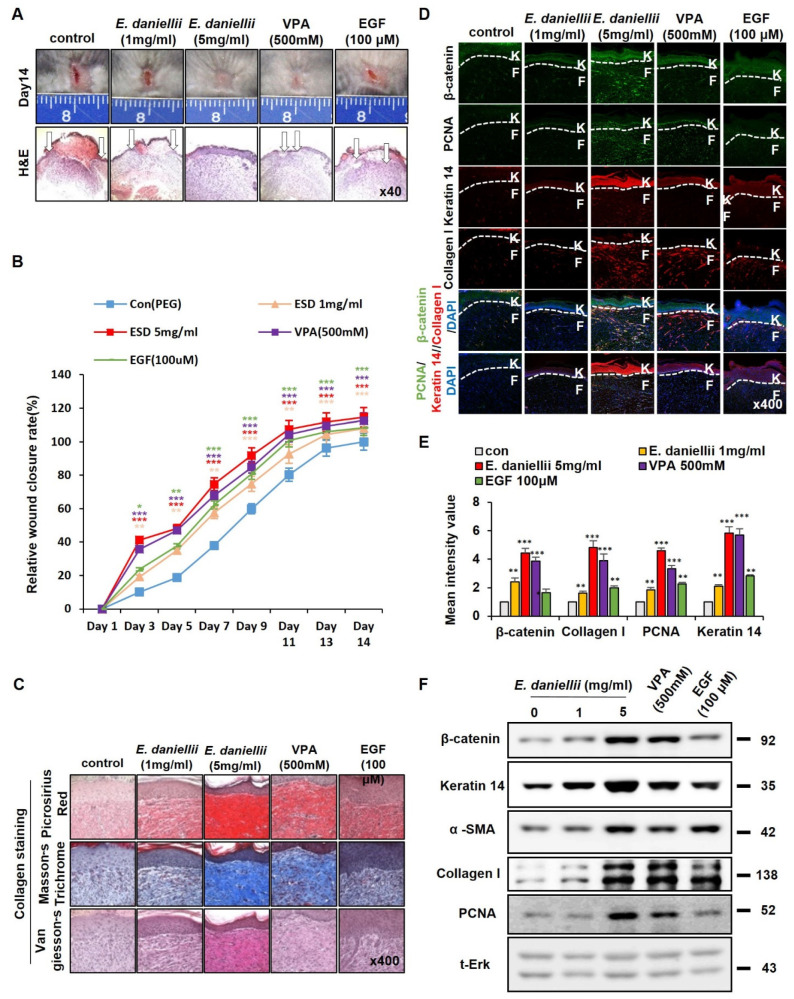
Effects of *E. daniellii* extract on cutaneous wound healing. Full-thickness wound (diameter = 1.0 cm) made on the back of 8-week-old male C3H mice, and vehicle (50% [*v*/*v*] ethanol, 30% water, and 20% propylene glycol), *E. daniellii* extract (1, 5 mg/mL), VPA (500 mM), or EGF (100 μM) were topically applied daily on the wounds up to 14 days. (**A**) Representative gross images of wounds at 14 days and H&E staining (lower panel). Arrows indicate wound edges. (**B**) Relative wound-closure rates were quantified as percent wound closure and closure rate of control group were considered as 100. Wound sizes were measured every 1, 3, 5, 7, 9, 12, and 14 days after creating the wound. (**C**–**F**) Wounded tissues excised from C3H mice 14 days postwounding and subjected to IHC analyses. (**C**) Representative images of Masson’s trichrome, picrosirius red, and van Gieson staining of wounded tissues (*n* = 6 mice/group) 14 days after wounding are shown (*n* = 3 independent experiments). (**D**) Representative images for IHC analyses of β-catenin, PCNA, keratin 14, and collagen I at wounds 14 days after wounding are shown. Dashed lines demarcate the epidermal–dermal boundary (*n* = 3 independent experiments). (**E**) Mean intensity values for Figure 2D (*n* = 3 independent experiments). (**F**) Immunoblotting analysis data for wounded tissue extracts obtained 14 days after wounding. Original magnification: (**A**), ×40; (**C**,**D**), ×400. Values are expressed as means ± SEM. * *p* < 0.05; ** *p* < 0.005; *** *p* < 0.0005, significantly different from vehicle, control, or as indicated.

To test the effect of the *E. daniellii* extract on cutaneous wounds in vivo, we performed an in vivo wound-healing assay using VPA and EGF [21] as positive controls. The wound-closure rate increased with enhanced re-epithelialization following treatment with *E. daniellii* extract. Both wound healing and re-epithelialization were mostly completed after 14 days of treatment with 5 mg/mL *E. daniellii* extract, and its effectiveness was equivalent to that of 500 mM VPA and higher than that of 100 μM EGF (Figure 3A,B). To further characterize the effects of *the E. daniellii* extract on wound healing related to Wnt/β-catenin signaling, wound-healing markers and β-catenin levels were monitored by IHC analyses in wound tissue sections harvested on day 14. Collagen deposition, a hallmark of wound healing, was increased by treatment with *the E. daniellii* extract, as confirmed by three independent collagen staining methods (Figure 3C). The level of β-catenin increased in both the keratinocytes and fibroblasts of skin tissues after treatment with the *E. daniellii* extract, and the effectiveness was greater than that shown by the VPA treatment (Figure 3D,E). The expression level of keratin 14, a marker for re-epithelialization and terminally differentiated keratinocytes, was increased, especially in newly formed epidermis, and collagen I, a marker for myo-fibroblast differentiation, was also synergistically increased in dermal fibroblasts after treatment with *E. daniellii* extract (Figure 3D,E). In addition, PCNA also increased in the neo-epidermis following treatment with *E. daniellii* extract (Figure 3D,E). The increase in β-catenin, keratin 14, α-SMA, collagen I, and PCNA levels by the *E. daniellii* extract treatment was further confirmed by Western blotting analyses performed with wound tissue sections harvested on day 14 (Figure 3F).

Taken together, *E. daniellii* extract accelerates in vitro wound healing and cutaneous wound healing in vivo via activation of the Wnt/β-catenin pathway.

### 3.3. Hesperidin Is an Active Component of E. daniellii Extract, and Accelerates the Migration of Human Keratinocytes and Dermal Fibroblasts via Activation of the Wnt/β-Catenin Signaling Pathway

**Figure 4:**
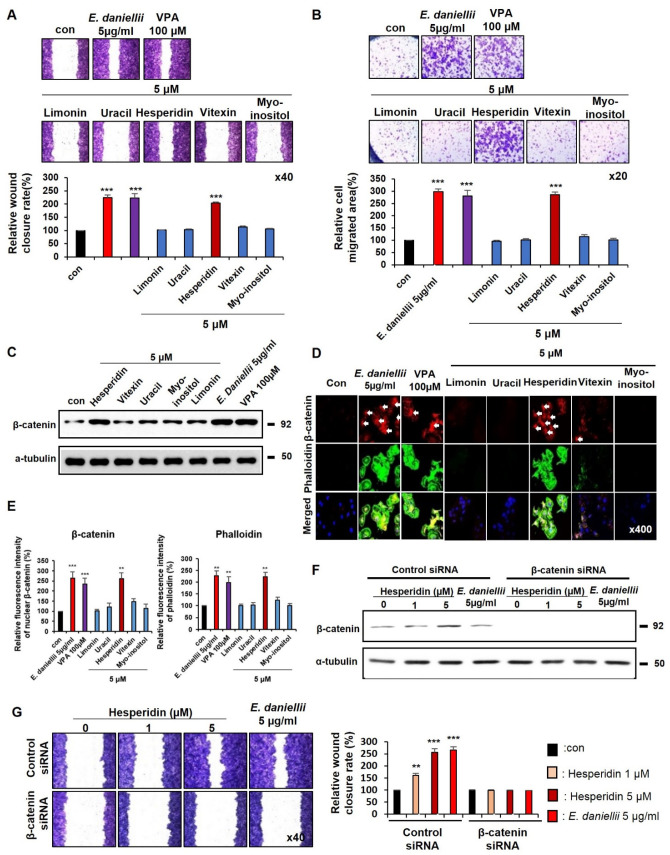
Effects of hesperidin on migration of human keratinocytes. (**A**–**D**) HaCaT keratinocytes were treated with vehicle (0.1% DMSO), 5 μM of *E. daniellii* components (hesperidin, vitexin, limonin, myo-inositol, uracil), *E. daniellii* extract (5 μg/mL), or VPA (100 μM) for 24 h. (**A**,**B**) The in vitro wound-healing and transwell assays were performed as described in the Materials and Methods section. (**A**) Representative images of in vitro wound-healing assay and quantitative measurement of relative wound-closure rate. (**B**) Representative images of transwell assay and quantitative measurement of the area of migrated cells. (**C**) Immunoblotting analysis data. (**D**) ICC staining for β-catenin and total phalloidin. Nuclei were counterstained with DAPI. Arrows indicate nucleus-localized β-catenin. (**E**) Quantitative measurements of intensities of β-catenin and phalloidin for the data from the experiments in Figure 3D (*n* = 10 taken in three different images). (**F**,**G**) HaCaT keratinocytes transfected with β-catenin siRNA or negative control for 12 h. After transfection, HaCaT keratinocytes treated with vehicle (0.1% DMSO), hesperidin (1, 5 μM), or *E. daniellii* extract (5 μg/mL) for 24 h. (**F**) Immunoblotting analysis data for transfected keratinocytes. (**G**) Representative images of in vitro wound-healing assay of transfected keratinocytes and quantitative measurement of wound-closure rate. Original magnification: (**A**,**B**), ×40; (**D**), ×400. Values are expressed as means ± SEM. ** *p* < 0.005; *** *p* < 0.0005, significantly different from vehicle, control, or as indicated.

**Figure 5:**
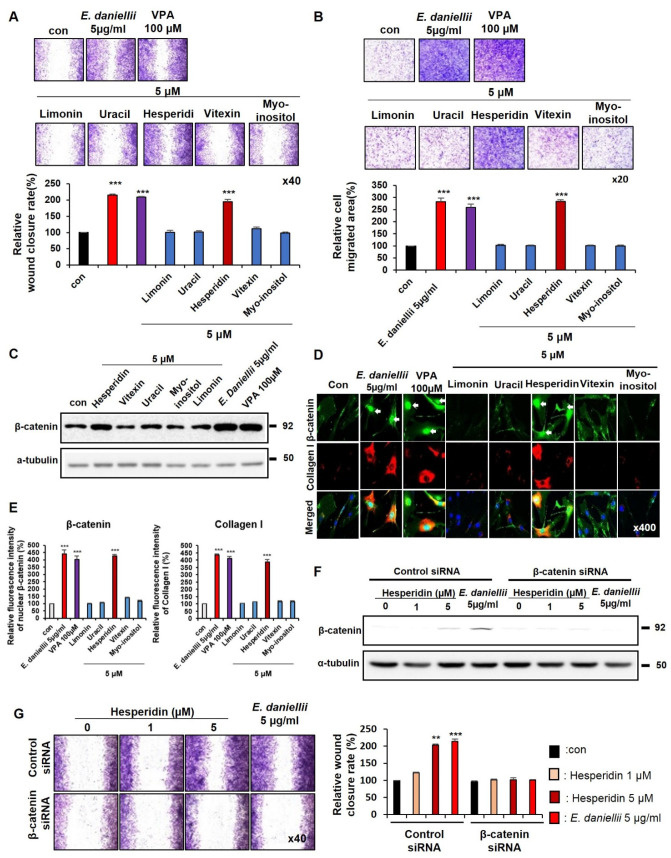
Effects of hesperidin on migration of human dermal fibroblasts. (**A**–**D**) Human dermal fibroblasts were treated with vehicle (0.1% DMSO), 5 μM of *E. daniellii* components (hesperidin, vitexin, limonin, myo-inositol, uracil), *E. daniellii* extract (5 μg/mL), or VPA (100 μM) for 24 h. (**A**,**B**) The in vitro wound-healing and transwell assays were performed as described in the Materials and Methods section. (**A**) Representative images of in vitro wound-healing assay and quantitative measurement of relative wound-closure rate. (**B**) Representative images of transwell assay and quantitative measurement of relative cell-migrated area. (**C**) Immunoblotting analysis data. (**D**) ICC staining for β-catenin and collagen I. Nuclei were counterstained with DAPI. Arrows indicate nucleus-localized β-catenin. (**E**) Quantitative measurements of intensities of β-catenin and Collagen I for the data from the experiments in Figure 5D (*n* = 10 taken in three different images). (**F**,**G**) Fibroblasts were transfected with β-catenin siRNA or negative control for 12 h. After transfection, fibroblasts were treated with vehicle (0.1% DMSO), hesperidin (1, 5 μM), or *E. daniellii* extract (5 μg/mL) for 24 h. (**F**) Immunoblotting analysis data for transfected fibroblasts. (**G**) Representative images of in vitro wound-healing assay of transfected fibroblasts and quantitative measurement of wound-closure rate. Original magnification: (**A**,**B**), ×40; (**D**), ×400. Values are expressed as means ± SEM. ** *p* < 0.005; *** *p* < 0.0005, significantly different from vehicle, control, or as indicated.

To identify the active component of the *E. daniellii* extract, we selected its ingredients, including hesperidin, vitexin, uracil, myo-inositol, and limonin [22] (Figure S2A). As confirmed by the TOPflash reporter assay, only hesperidin increased Wnt/β-catenin signaling activity in a dose-dependent manner (Figure S2B). We first performed in vitro wound-healing and transwell assays in both keratinocytes and fibroblasts to identify the active ingredient of *E. daniellii* that enhances cell migration. Among the components, only hesperidin increased the migration of keratinocytes and fibroblasts, similar to the *E. daniellii* extract (Figure 4A,B and Figure 5A,B).

The ability of hesperidin to increase cell migration correlates with a specific increase in β-catenin levels, as confirmed by Western blotting in keratinocytes and fibroblasts (Figure 4C and Figure 5C). Hesperidin specifically induced the nuclear accumulation of active β-catenin and increased phalloidin and collagen I levels in human keratinocytes and dermal fibroblasts (Figure 4D,E and Figure 5D,E). As observed in the *E. daniellii* extract-treated cells, cell migration enhancement and β-catenin induction by hesperidin were mostly abolished by β-catenin knockdown in both keratinocytes and fibroblasts, indicating that hesperidin accelerates migration in vitro via the activation of Wnt/β-catenin signaling (Figure 4F,G and Figure 5F,G). Hesperidin showed no significant toxicity in keratinocytes, dermal fibroblasts, or primary neural stem cells (Figure S3). We performed HPLC analysis of *E. daniellii* extract based on UV absorbance in 254 nm wavelength to confirm the quantity and quality of selected components (Figure S4). We confirmed that portion of hesperidin was the highest among the selected components (Figure S4A). We also confirmed no significant changes in chemical profile of *E. daniellii* extract throughout experiments (Figure S4B).

These results showed that the active ingredient of *E. daniellii* hesperidin stimulates the migration of keratinocytes and fibroblasts via the activation of the Wnt/β-catenin signaling pathway in vitro.

### 3.4. Hesperidin Promotes Cutaneous Wound Healing In Vivo

**Figure 6:**
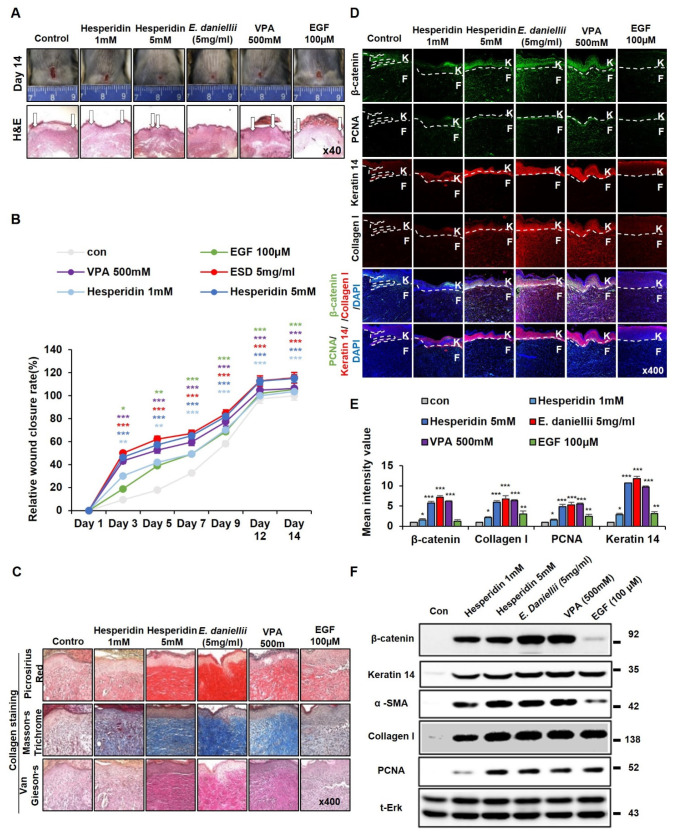
Effects of hesperidin on cutaneous wound healing. Full-thickness wound (diameter = 1.0 cm) were made on the back of 8-week-old male C3H mice, and vehicle (50% ethanol, 30% water, and 20% propylene glycol), hesperidin (1, 5 mM), *E. daniellii* extract (5 mg/mL), VPA (500 mM), or EGF (100 μM) were topically applied daily on the wounds for 14 days. (**A**) Representative gross images of wounds on day 14 and H&E staining (lower panel). Arrows indicate wound edges. (**B**) Wound sizes and relative wound-closure rates were measured as described in Figure 2B. (**C**–**F**) Wounded tissues excised from C3H mice 14 days post-wounding and subjected to IHC analysis. (**C**) Representative images of Masson’s trichrome, picrosirius red, and van Gieson staining of wounded tissues (*n* = 6 mice/group) 14 days after wounding are shown (*n* = 3 independent experiments). (**D**) Representative images of β-catenin, PCNA, keratin 14, and collagen I of wounds 14 days after wounding are shown. Dashed lines demarcate the epidermal–dermal boundary (*n* = 3 independent experiments). (**E**) Mean intensity values for Figure 4D (*n* = 3 independent experiments). (**F**) Immunoblotting analysis data for wounded tissue extracts obtained 14 days after wounding. Original magnification: (**A**), ×40; (**C**,**D**), ×400. Values are expressed as means ± SEM. * *p* < 0.05; ** *p* < 0.005; *** *p* < 0.0005, significantly different from vehicle, control, or as indicated.

To identify the cutaneous wound-healing effect of hesperidin, we performed an in vivo wound-healing assay, as described above. Re-epithelialization and wound closure were mostly completed by treatment with 5 mM hesperidin, as shown by the effects of treatment with 5 mg/mL *E. daniellii* extract (Figure 6A,B). The effect of the *E. daniellii* extract on collagen deposition was largely increased by treatment with hesperidin in the wound tissue (Figure 6C). In addition, hesperidin significantly increased β-catenin levels and wound-healing markers, as observed in *E. daniellii* (Figure 6D,E). Moreover, hesperidin dose-dependently increased the protein levels of β-catenin, keratin 14, α-SMA, collagen I, and PCNA compared with the vehicle-treated group, as analyzed by Western blotting (Figure 6F).

Overall, hesperidin is the active component of *the E. daniellii* extract and accelerates wound healing by activating the Wnt/β-catenin signaling pathway.

## 4. Conclusions

With the expansion of the medical and cosmetic markets, there is a growing need for new agents to overcome the limitations of current wound-healing agents [4]. Current wound-healing agents, such as antibiotic-based agents, can result in cellular and organ toxicity [5], and growth-factor-based agents have a short half life, high cost, limited effects on re-epithelialization, and poor absorption rate [23]. Stem cell therapy is attractive as a regenerative therapy but has limitations in administration, quality control, cost, and safety issues [24]. Targeting the signaling pathways involved in tissue regeneration has emerged as a new approach for the development of wound-healing agents [8].

The transforming growth factor beta (TGF-β), Notch, Hedgehog, and Wnt/β-catenin pathways are known to be involved in tissue regeneration and are required for wound healing and skin development [25]. Especially, the Wnt/β-catenin pathway is a major signaling pathway for stem cell activation and induces multiple factors involved in wound healing such as Collagen-1, WISP1, Keratin-14, EGFR, and VEGF [8]. Therefore, the Wnt/β-catenin pathway is considered an attractive target for the development of wound-healing agents.

In this study, we investigated the effects of *E. daniellii* extract on wound healing using both in vitro and in vivo analyses. The *E. daniellii* extract enhanced cell migration without causing significant cellular cytotoxicity, with an increase in the levels of stress fibers and myo-fibroblast differentiation markers in human keratinocytes and dermal fibroblasts. Treatment with *E. daniellii* extract, the hallmark factors for wound healing, including the deposition of Collagen-1 and inductions of keratin 14 and α-SMA, was increased in a murine wound-healing model with the acceleration of the proliferation and re-epithelialization of the wound tissues.

Hesperidin, a flavanone glycoside, was identified as a specific active component of the *E. daniellii* extract and enhanced wound healing in both in vitro and in vivo system. We also confirmed that the wound-healing effects of both *E. daniellii* extract and hesperidin were mediated via the Wnt/β-catenin signaling pathway, as shown by the siRNA-mediated β-catenin knockdown. Moreover, the *E. daniellii* extract did not show any significant cellular toxicity.

Our data showed that *E. daniellii* extract and its active component, hesperidin, promote human keratinocyte and dermal fibroblast migration, and accelerate re-epithelialization in a mouse wound model via the activation of the Wnt/β-catenin signaling pathway. Since plant materials are considered to be suitable candidates for therapeutics, our findings suggest that *E. daniellii* extract and its active component hesperidin could have the potential to be used as bio-agents for wound healing.

## Data Availability

All data associated with this study are available from the corresponding author upon reasonable request.

## Supplementary Materials

The following supporting information can be downloaded at: https://www.mdpi.com/article/10.3390/molecules27207134/s1. Figure S1: TOPFlash reporter activity, cellular toxicity tests, and concentration range test of *E. daniellii* extract; Figure S2: Structure of *E. daniellii* ingredients and TOPFlash reporter activity of hesperidin; Figure S3: Cellular toxicity tests of hesperidin; Figure S4: HPLC analysis of *E. daniellii* extract.

## Sample Availability

Samples of the compounds are not available from the authors.
