# Supplementary material for: Euodia daniellii Hemsl. Extract and Its Active Component Hesperidin Accelerate Cutaneous Wound Healing via Activation of Wnt/β-Catenin Signaling Pathway
Source: Molecules. 2022 Oct 21;27(20):7134. doi: 10.3390/molecules27207134 (PMC9608813; doi:10.3390/molecules27207134)
Supplement: Supplementary file 1 [file molecules-27-07134-s001.zip › molecules-1947831-supplementary.pdf]

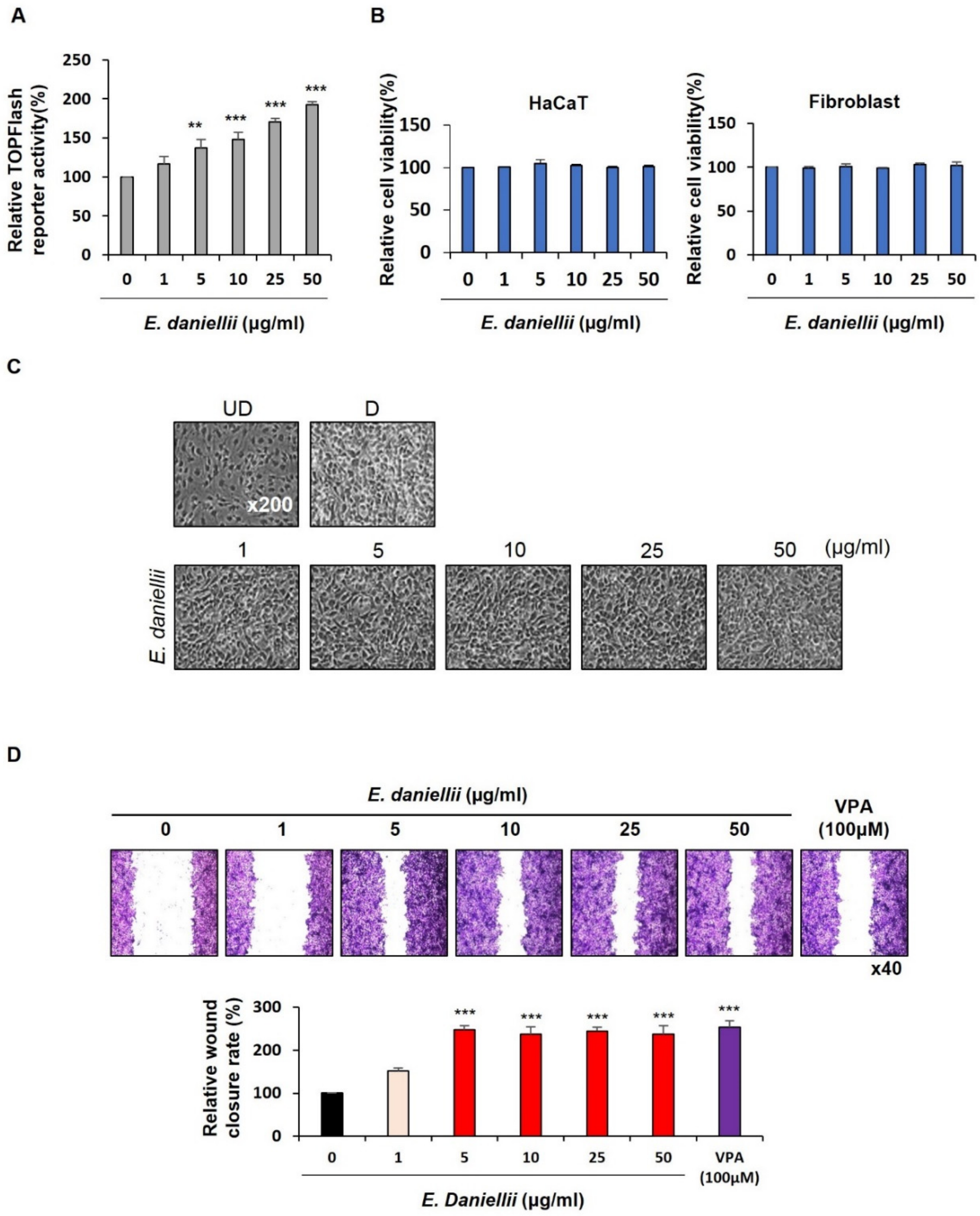

Supplementary Figure S1. TOPFlash reporter activity, cellular toxicity tests, and concentration range test of *E. daniellii* extract. (A) TOPFlash reporter activity of HEK293 cells treated with vehicle (0.1% DMSO), or shown concentrations of *E. daniellii* extract for 24 h (n = 3). (B) Cell viability of HaCaT keratinocytes and human dermal fibroblasts treated with vehicle (0.1% DMSO), or shown concentration of *E. daniellii* extract for 24 h (n = 3). (C) Brightfield images of cultured primary neural stem cell morphology treated by shown concentration of *E. daniellii* extract. (D) HaCaT keratinocytes were treated with vehicle (0.1% [*v*/*v*] DMSO), *E. daniellii* extract (1, 5, 10, 25, 50 μg/mL), or VPA (100 μM) for 24 h. The in vitro wound-healing assay was performed as described in the Materials and Methods section. Representative images of in vitro wound-healing assay (upper) and quantitative measurement of relative wound-closure rate (lower). Original magnification: (C), x200; (D), x40. Values are expressed as means ± SEM. **, P < 0.005; ***, P < 0.0005, significantly different from vehicle, control or as indicated.

A

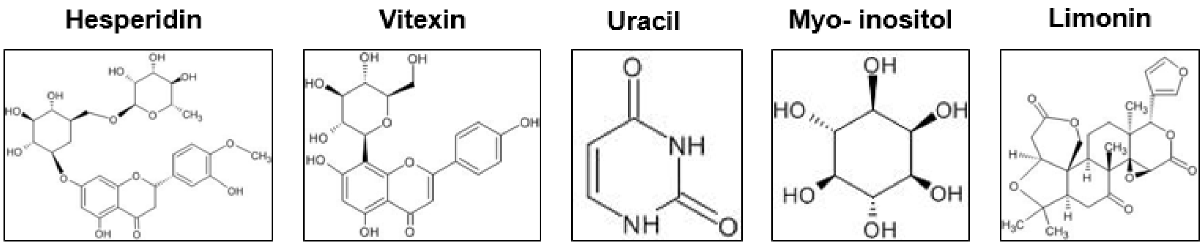

B

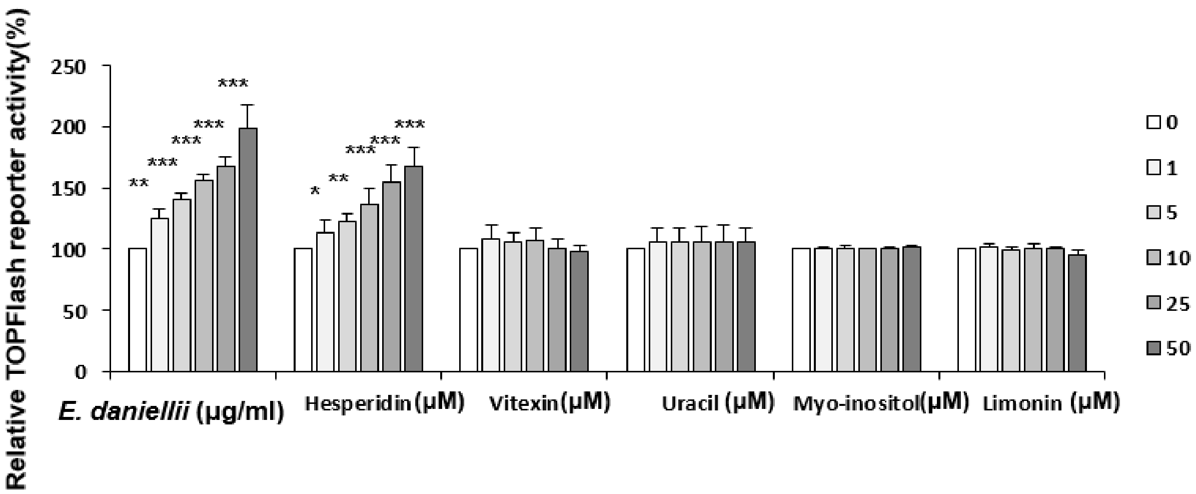

Supplementary Figure S2. Structure of *E. daniellii* ingredients and TOPFlash reporter activity of hesperidin (A) Structure of *E. daniellii* ingredients. (B) TOPFlash reporter activity of HEK293 cells treated with vehicle (0.1% DMSO), or shown concentrations of hesperidin, vitexin, limonin, myo-inositol, uracil for 24 h (n = 3). Values are expressed as means ± SEM. *, $P < 0.05$; **, $P < 0.005$; ***, $P < 0.0005$, significantly different from vehicle, control or as indicated.

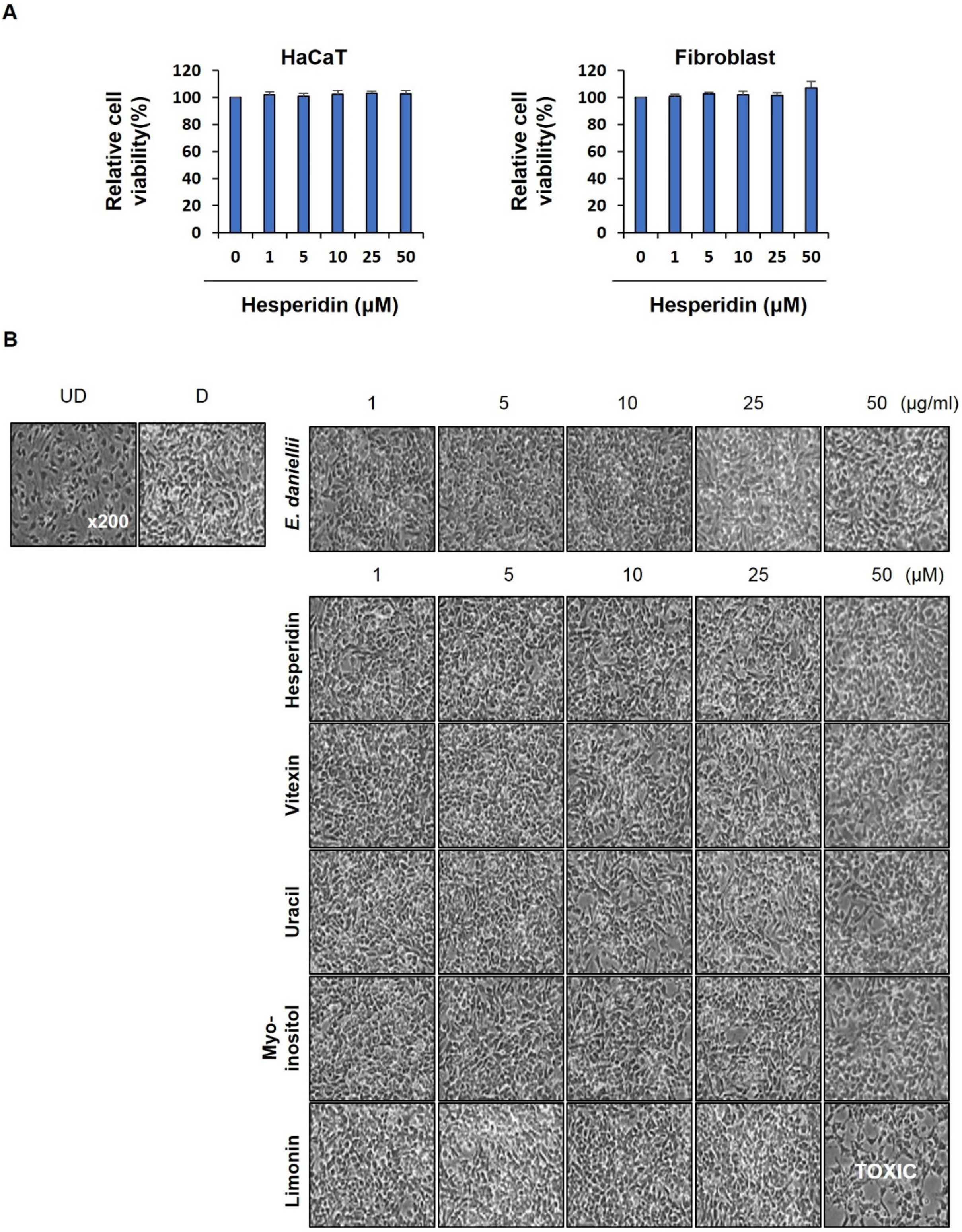

Supplementary Figure S3. Cellular toxicity tests of hesperidin. (A) Cell viability of HaCaT keratinocytes and human dermal fibroblasts treated with vehicle (0.1% DMSO), or shown concentration of hesperidin for 24 h (n = 3). (B) Brightfield image of cultured primary neural stem cell morphology treated by shown concentration of hesperidin for 24 h. Original magnification: (B) x200. Values are expressed as means ± SEM.

A

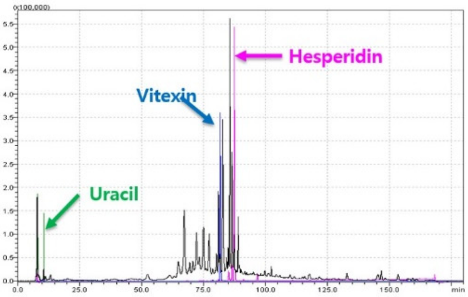

	Retention time	Percentage in Extract
**Hesperidin**	87.447	2.633 %
**Vitexin**	81.774	1.620 %
**Uracil**	10.559	0.355 %
**Limonin**	ND	-
**Myo-inositol**	ND	-

B

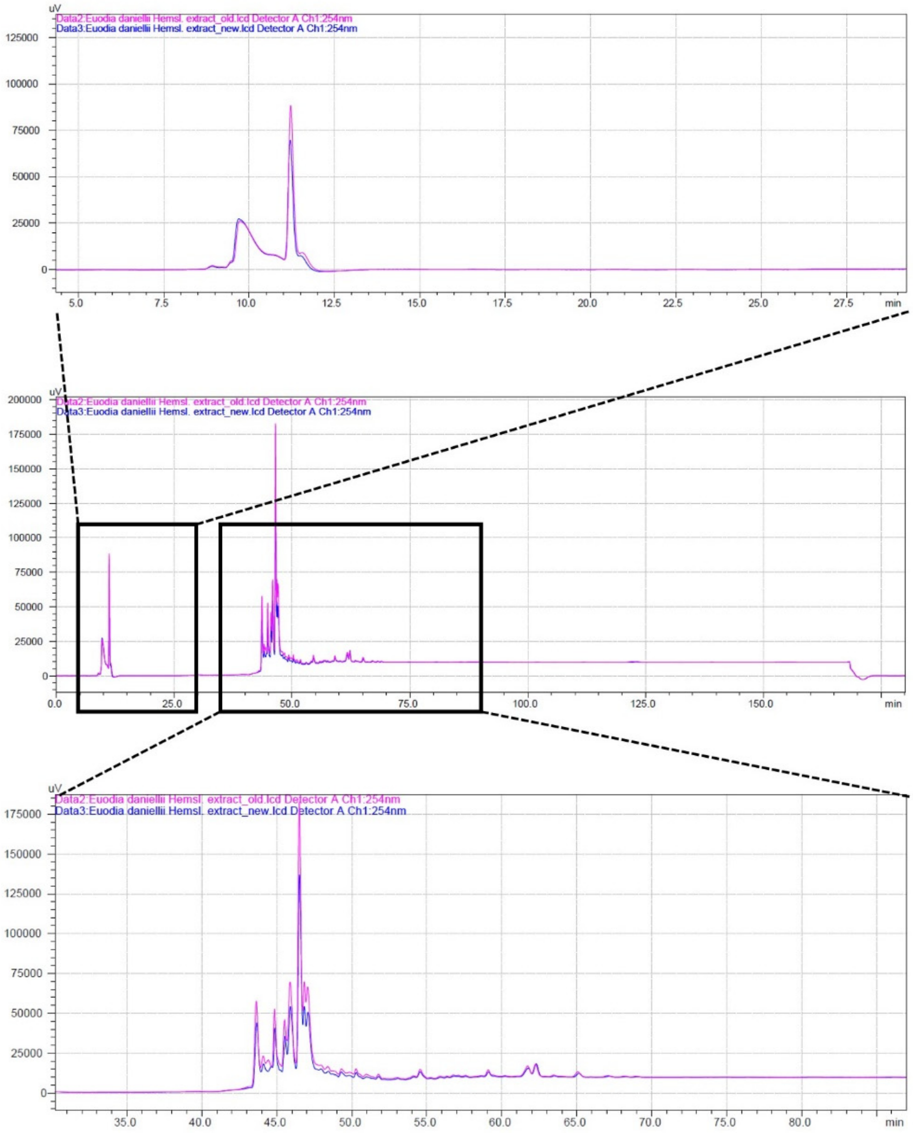

Supplementary Figure S4. HPLC analysis of *E. daniellii* extract. (A,B) HPLC analysis was performed as described in the Materials and Methods section. (A) The composition of vitexin, uracil and heperidin in *E. daniellii* extract. (B) Chemical profile comparison of existing *E. daniellii* extract (*E. daniellii* extract old) and newly purchased *E. daniellii* extract (*E. daniellii* extract new).
